# Supplementary material for: Transient polymorphisms in parental care strategies drive divergence of sex roles
Source: Nat Commun. 2023 Oct 26;14:6805. doi: 10.1038/s41467-023-42607-6 (PMC10603145; doi:10.1038/s41467-023-42607-6)
Supplement: Supplementary file 1 — Supplementary Information [file 41467_2023_42607_MOESM1_ESM.pdf]

Supplementary information

Transient polymorphisms in parental care strategies drive divergence of sex roles

Xiaoyan Long[1, 2] and Franz J. Weissing[1*]

[1]Groningen Institute for Evolutionary Life Sciences, University of Groningen, Groningen 9747AG, The Netherlands

[2]Present address: Institute of Biology I, University of Freiburg, Freiburg im Breisgau 79104, Germany

[*]To whom correspondence should be addressed. Email: f.j.weissing@rug.nl

This PDF file includes:

Supplementary Figure 1. Diagram illustrating the complete life cycle in our model.

Supplementary Figure 2. Evolutionary branching of parental care strategies.

Supplementary Figure 3. Effect of initial conditions on the evolution of parental sex roles.

Supplementary Figure 4. Evolution of parental roles when parental synergy is large.

Supplementary Figure 5. Effect of population size on the timing of evolutionary transitions.

Supplementary Figure 6. Effect of mortality rates on the timing of evolutionary transitions.

Supplementary Figure 7. Evolutionary transitions via transient polymorphisms.

Supplementary Figure 8. Transitions between alternative mating and caring strategies.

Supplementary Figure 9. Effect of sex-biased pre-mating investment and initial care conditions on parental sex roles.

Supplementary Figure 10. Rescaled version of the Fromhage & Jennions (2016) model.

Supplementary Figure 11. Evolution of sex-biased care in the rescaled FJ model.

Supplementary Figure 12. Pairwise Invasibility Plot of the rescaled FJ model.

SI References

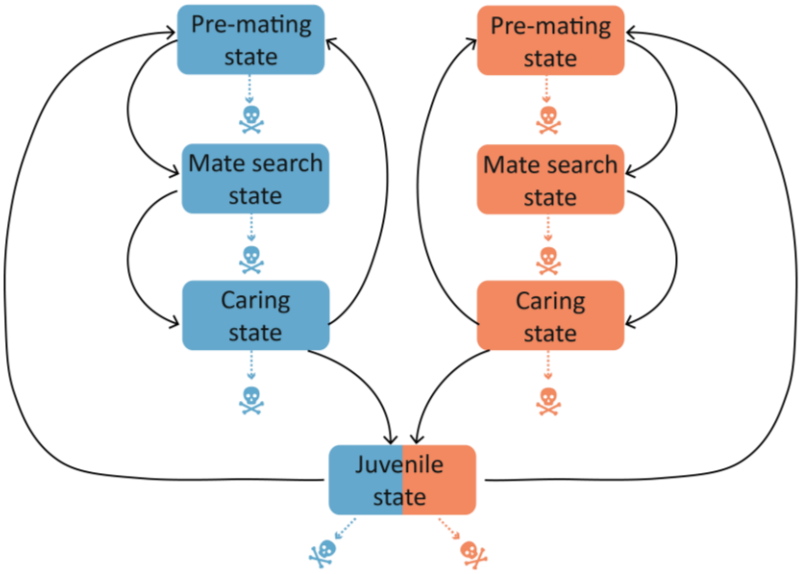

Supplementary Figure 1. Diagram illustrating the complete life cycle in our model. Individuals have the same life cycle as shown in Fig. 1, but with a 'pre-mating' state. Instead of immediately entering the mate search state, offspring spend a fixed time period (which is set to zero in Fig. 1) in the 'pre-mating state. Furthermore, once the parents have finished caring for their offspring, they switch to the pre-mating state, from where the whole cycle repeats itself. Mortality can occur in any state, including the pre-mating state, and dead individuals are removed from the population. The colour red indicates females, and the colour blue indicates males.

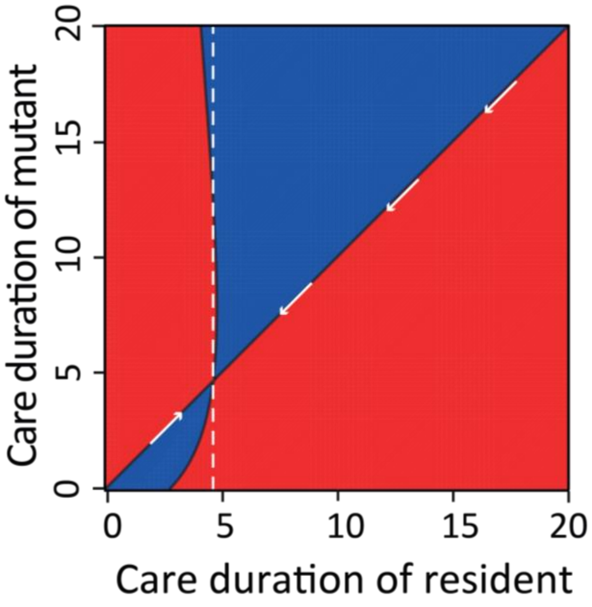

Supplementary Figure 2. Evolutionary branching of parental care strategies. The graph shows a 'Pairwise Invasibility Plot (PIP)' of the baseline version of our model (random mating, no differences in life histories between the sexes). We restrict attention to egalitarian care ($T_f = T_m$), allowing us to conduct a one-dimensional analysis. A PIP illustrates which mutant strategies can invade when rare in a given resident population. As explained in detail in Geritz *et al.*[1], the *x*-axis depicts all possible care durations of a resident population, while the care durations of mutants are represented on the *y*-axis. The red area of the plot corresponds to mutant-resident combinations where the mutant has a higher fitness than the resident and, hence, can invade the resident population. Here, fitness is calculated as in Fromhage and Jennions[2]. The blue area indicates those mutant-resident combinations where the mutant has a lower fitness than the resident and is selected against. The separating black lines corresponds to situations where mutants and residents have the same fitness. The two equal-fitness lines intersect at the value $T^* = 4.67$, which is a so-called 'Evolutionarily Singular Strategy'. The white arrows indicate that this strategy is 'convergence stable': in the course of evolution, the resident population is shifted toward T^*. The dashed vertical line lies in the red region (at least for mutants close to T^*), implying that there are mutants that can invade the resident T^*. This means that T^* is *not* evolutionarily stable. A configuration like this (convergence to an evolutionarily unstable strategy) is called a 'branching point', because it indicates that directional selection (toward T^*) switches to disruptive selection (once T^* is reached). When the population would remain constrained to egalitarian care, a dimorphic population would result, where part of the population would care less than $T^* = 4.67$ while another part would care more than this value. If sex differentiation in care is possible, it is to be expected that each of the 'branches' gets associated with one of the two sexes (e.g., low-care might get associated with the female sex and high-care with the male sex) (see ref. 3). A *Mathematica* file with the implementation of the PIP is available via the link in the code availability statement in the main text.

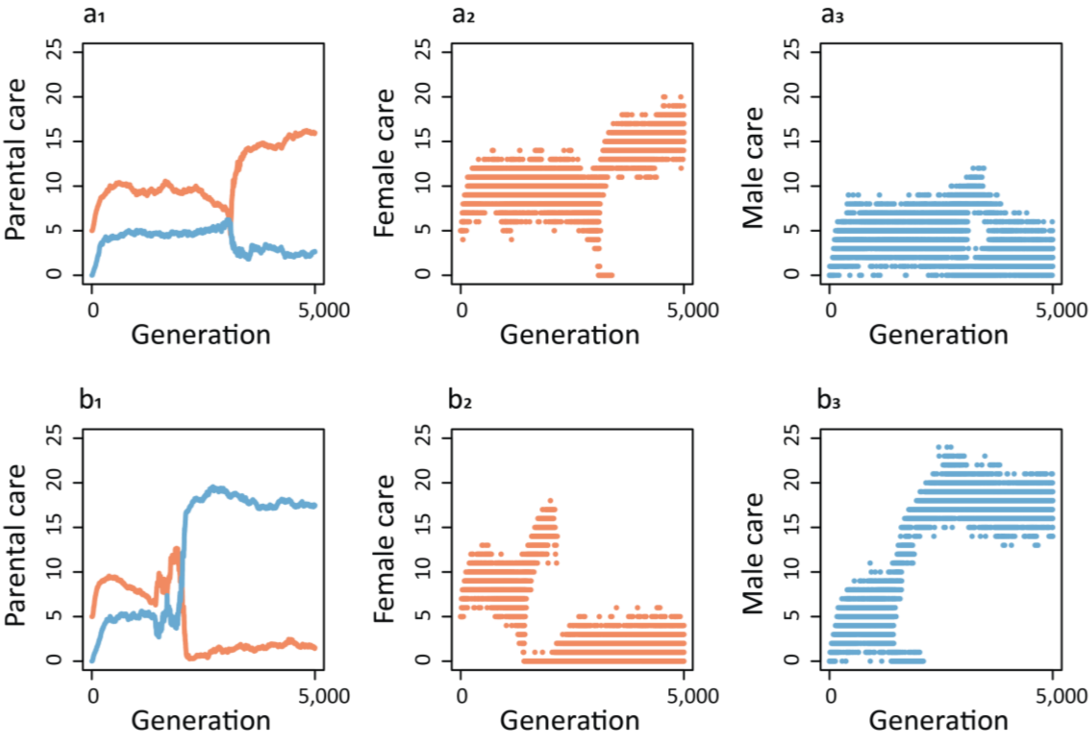

Supplementary Figure 3. Effect of initial conditions on the evolution of parental sex roles. In the main text, we focus on the initial conditions $T_f = T_m = 20$, corresponding to high-level egalitarian biparental care. The same evolutionary patterns as in Figs. 2d, 2c and 3 were observed for all simulations starting with egalitarian biparental care, irrespective of the initial level of care. The graphs above illustrate that the same holds in the case of asymmetric initial conditions, with the difference that the resulting sex-role equilibria are no longer equally likely, but that the sex that initially cares more is (much) more likely to end up in the role of the caring sex. The graphs above show two representative evolutionary trajectories for simulations initialised with a low level of female-only care ($T_f = 5$ and $T_m = 0$). Lines show the average care levels of females (red) and males (blue) in the population, while dots represent individual care levels. The simulation in (**a**) corresponds to one of the blue-coloured lines in Fig. 2b. All 100 simulations first evolved towards a situation where males and females cared, on average, at a level of $T_f = T_m = 5$, at which polymorphism emerged in both sexes, which in turn collapsed, eventually resulting in one of the two stable equilibria shown in Fig. 2b. In 96 of the 100 simulations, (**a$_1$**) strongly female-biased care evolved through a short period of polymorphisms in (**a$_2$**) female and (**a$_3$**) male care strategies. In 4 of 100 simulations, (**b$_1$**) strongly male-biased care evolved, also via transient polymorphisms in (**b$_2$**) female and (**b$_3$**) male care strategies. The asymmetry in outcomes can be explained as follows. When approaching the "branching point" ($T_f = T_m = 5$), the initial asymmetry $T_f > T_m$ leaves its mark on the emerging polymorphisms: the two "branches" are typically further apart in females than in males (as in (**a$_2$**) and (**a$_3$**)). As a consequence, the zero-care females have a lower reproductive success than the zero-care males. This makes the collapse of the zero-care branch more likely in females than in males, resulting in the reported pattern of evolutionary outcomes.

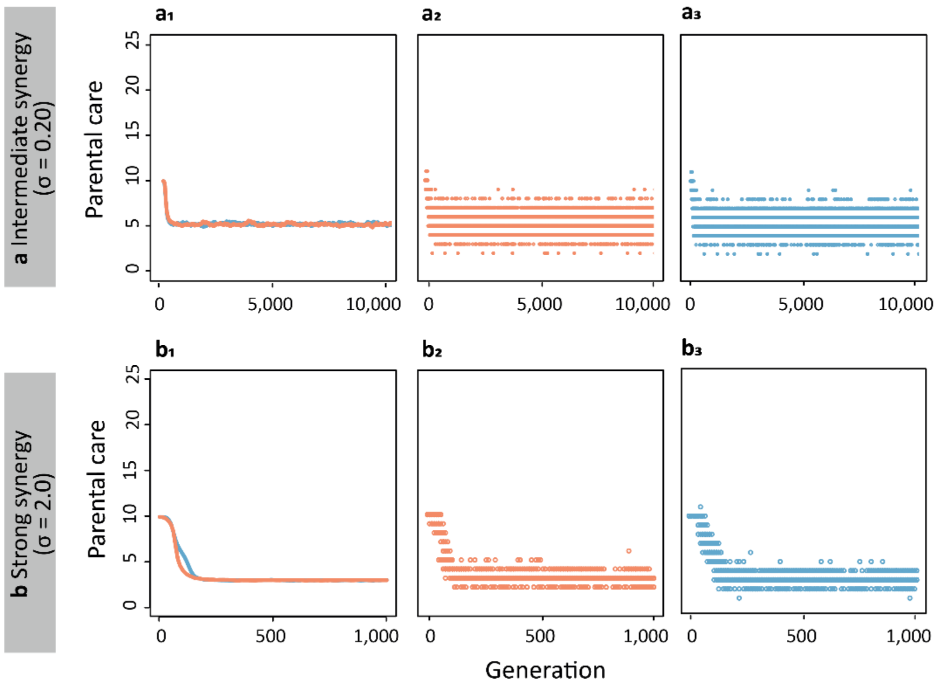

Supplementary Figure 4. Evolution of parental roles when parental synergy is large. Representative simulations for the case that the synergistic effects of the parents on offspring survival are relatively large. Lines show the average care levels of females (red) and males (blue) in the population, while dots represent individual care levels. **(a)** In case of intermediate synergy ($\sigma = 0.20$), **(a_1)** evolution leads to egalitarian care equilibrium. However, diverse care strategies coexist in **(a_2)** females and **(a_3)** males. Total care $T_f + T_m + \sigma T_f T_m$ is considerably smaller than $B = 20$, the value maximising the marginal benefit of care in our model. **(b)** In case of strong synergy ($\sigma = 2.0$), **(b_1)** the evolving egalitarian-care equilibrium exhibits relatively little variation in **(b_2)** females and **(b_3)** males, and total care now matches $B = 20$.

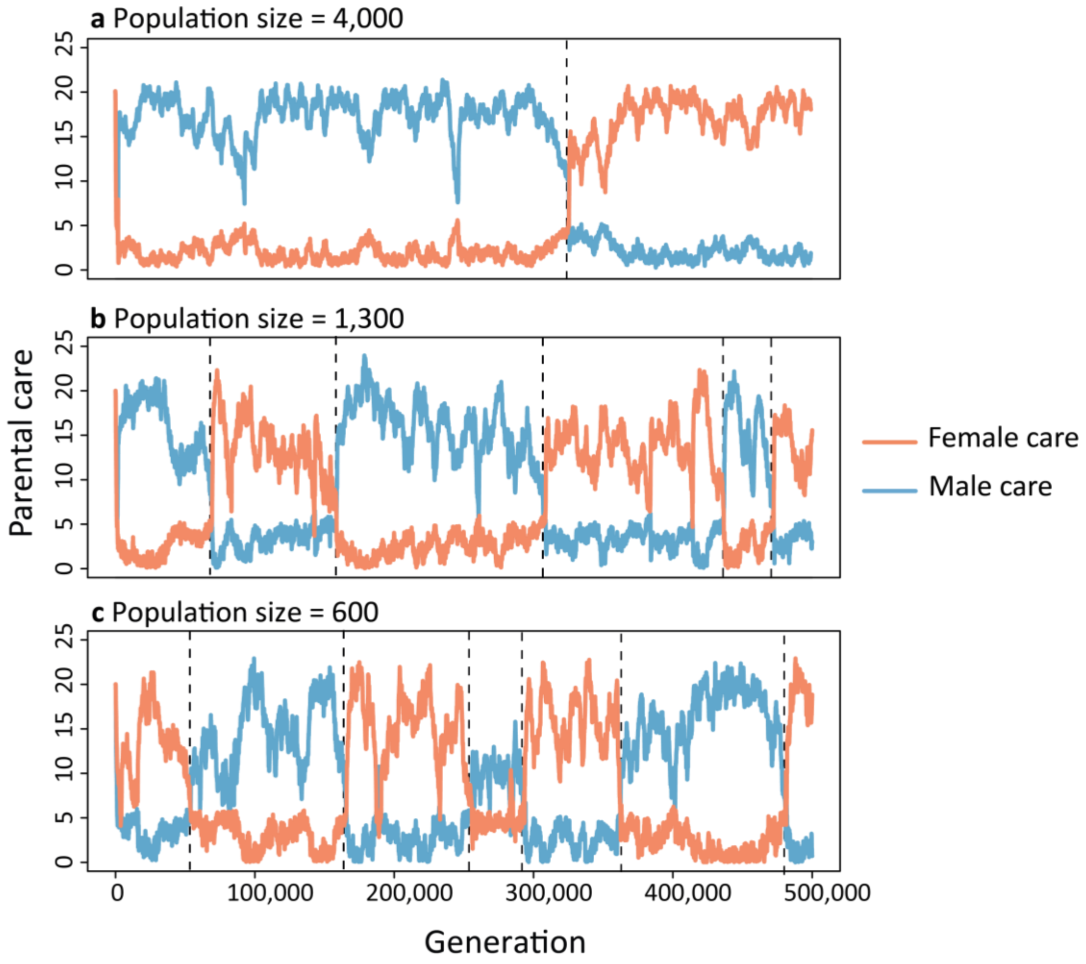

Supplementary Figure 5. Effect of population size on the timing of evolutionary transitions. As shown in Fig. 5 of the main text, parental roles are evolutionarily labile in that spontaneous transitions occur from one parental-care equilibrium to the other. As explained in the main text, the mean time between transitions depends on the duration of the pre-mating period (affecting the strength of selection) and on population size (affecting genetic drift). The three panels illustrate, for simulations without pre-mating period, how the number of transitions within a fixed period of 500,000 generations increases with a decrease of population size. **(a)** 2,000 females and 2,000 males: one transition; **(b)** 650 males and 650 males: five transitions; **(c)** 300 females and 300 males: six transitions. Population sizes fluctuated and were regulated by changing model parameter γ (see Methods). Lines show the average care levels of females (red) and males (blue) in the population.

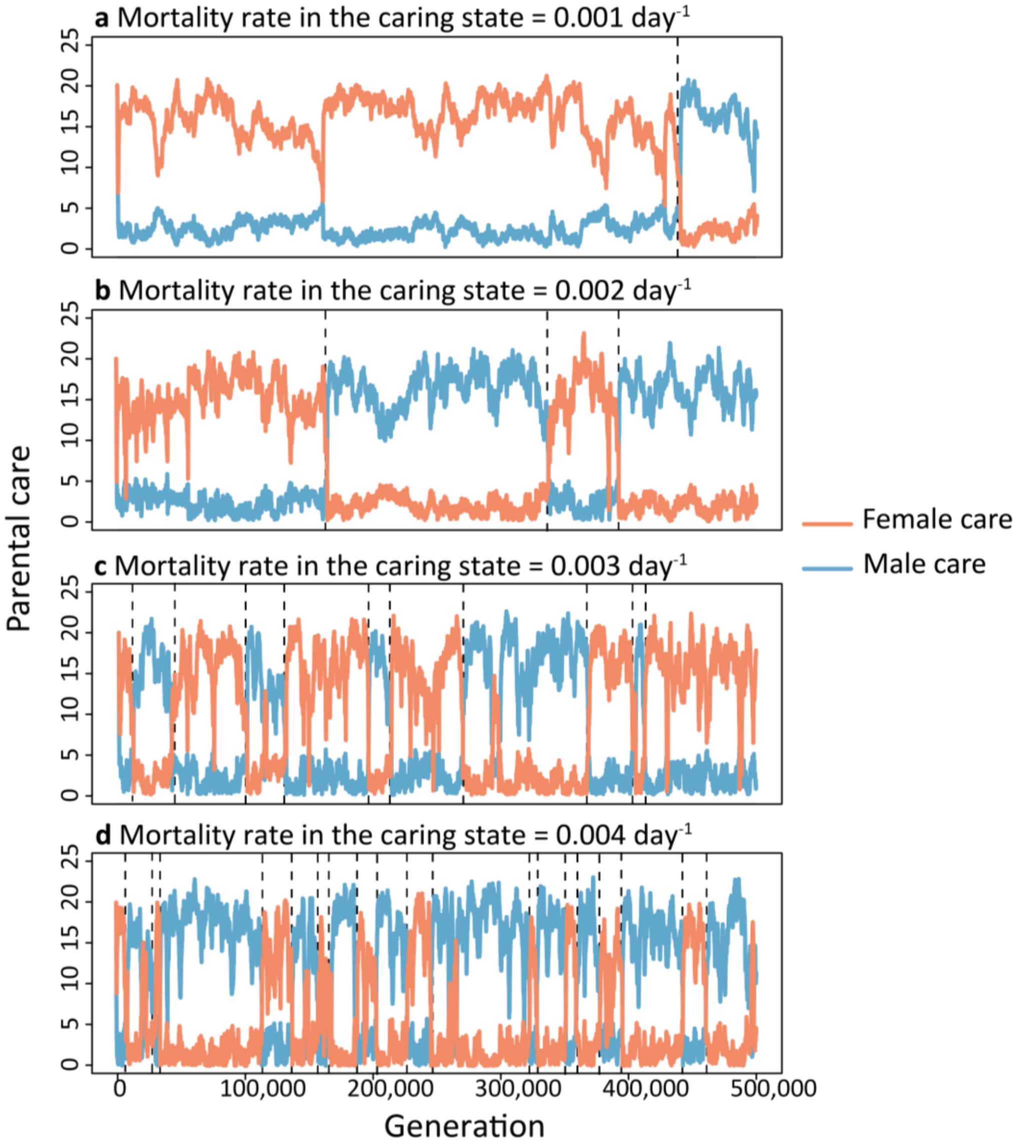

Supplementary Figure 6. Effect of mortality rates on the timing of evolutionary transitions. Mortality rates affect the average time between transitions via influencing genetic drift. The graphs show that the number of transitions increases as mortality rates of males and females in the caring state increase. In each simulation, mortality rates of each sex in the juvenile, pre-mating and mating were set to 0.001 day^{-1}, while the mortality rate in the caring state was **(a)** 0.001 day^{-1}: one transitions; **(b)** 0.002 day^{-1}: three transitions; **(c)** 0.003 day^{-1}: ten transitions; **(d)** 0.004 day^{-1}: nineteen transitions. Lines show the average care levels of females (red) and males (blue) in the population.

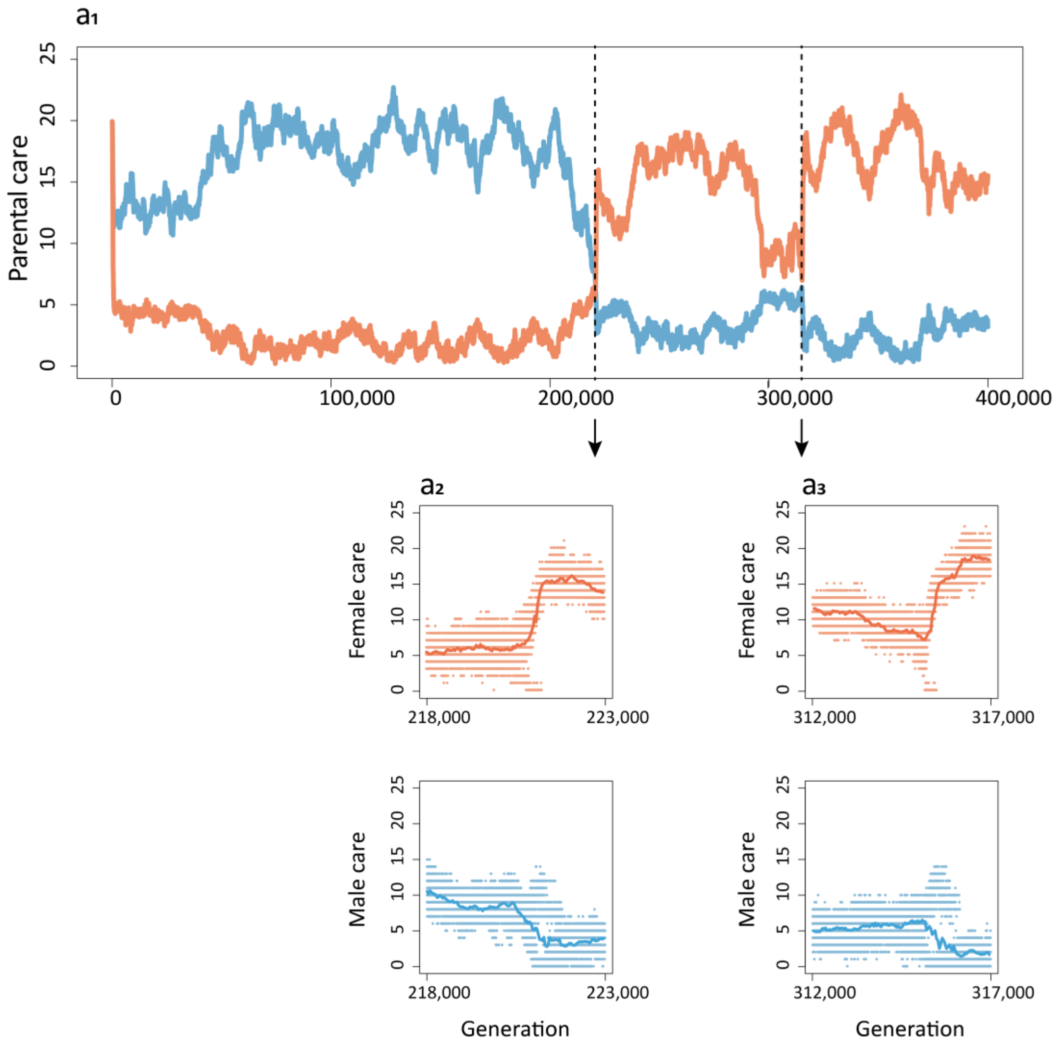

Supplementary Figure 7. Evolutionary transitions via transient polymorphisms. Switches between the female-care equilibrium and the male-care equilibrium occurred via a short period of polymorphism in the care levels of both sexes. The graphs illustrate this for a replicate simulation of the simulation depicted in Fig. 5 of the main text. As in Fig. 5, the time trajectory in (**a_1**) again shows rapid switches from one equilibrium to the other one. Both male care equilibrium and female care equilibrium are stable, but the care levels of both sexes fluctuate to some extent. During these fluctuations, once the care levels of the both sexes reach 5, the population converges to the evolutionary branching point ($T_f = T_m = 5$), where transient polymorphisms occur in both sexes (around generation 220,000, see (**a_2**) and generation 315,000, see (**a_3**)). Upon the emerging of transient polymorphisms in both sexes, the population either converges to the alternative equilibrium (as in a_2) or it reverts back to the previous equilibrium (as in a_3). Lines show the average care levels of females (red) and males (blue) in the population, while dots represent individual care levels.

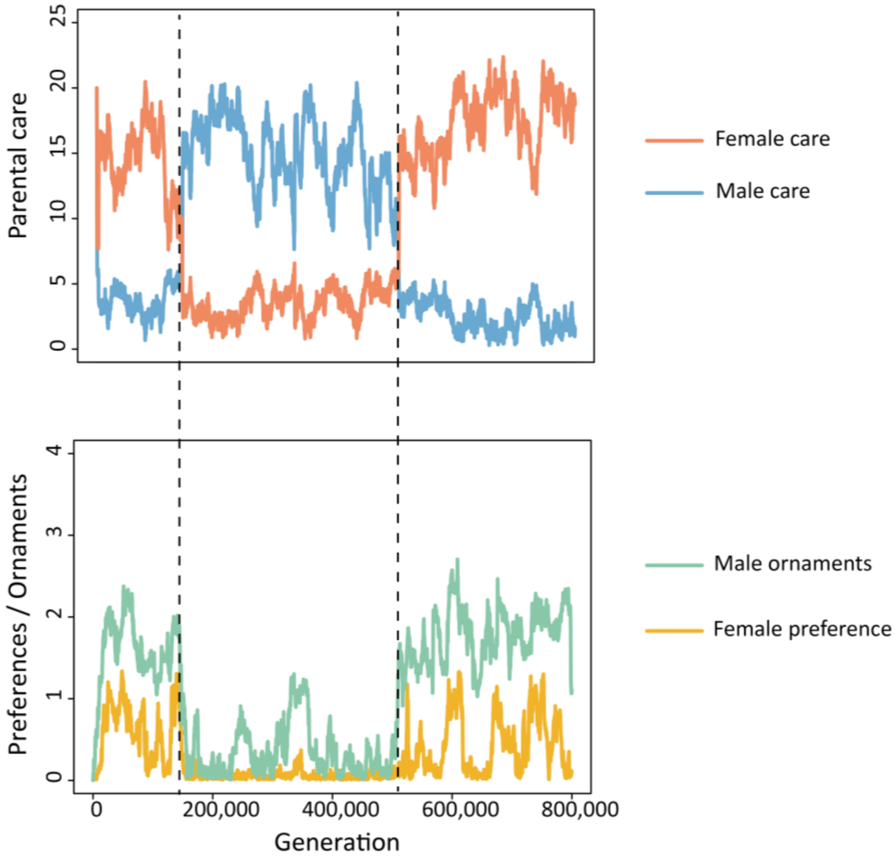

Supplementary Figure 8. Transitions between alternative mating and caring strategies. As shown in Fig. 6 of the main text, the joint evolution of mating and parental care strategies leads to one of two equilibria: male care in the absence of female choosiness and female care, female care associated with a female preference for ornamented males and costly male ornamentation. The simulation demonstrates that also these combined mating and parental roles are evolutionarily labile in that spontaneous transitions occur from one equilibrium to the other. Epochs with female choosiness and female-biased care (here: first 180,000 generations, last 300,000 generations) alternate with epochs with random mating (= no female preference) and male-biased care (here: generations 180,000 till 500,000). A more detailed analysis revealed that the change in female preferences was always preceded by a change in the parental care strategies of the two sexes. Lines show the average female care level (red), male care level (blue), female preferences (yellow) and male ornaments (green) in the population.

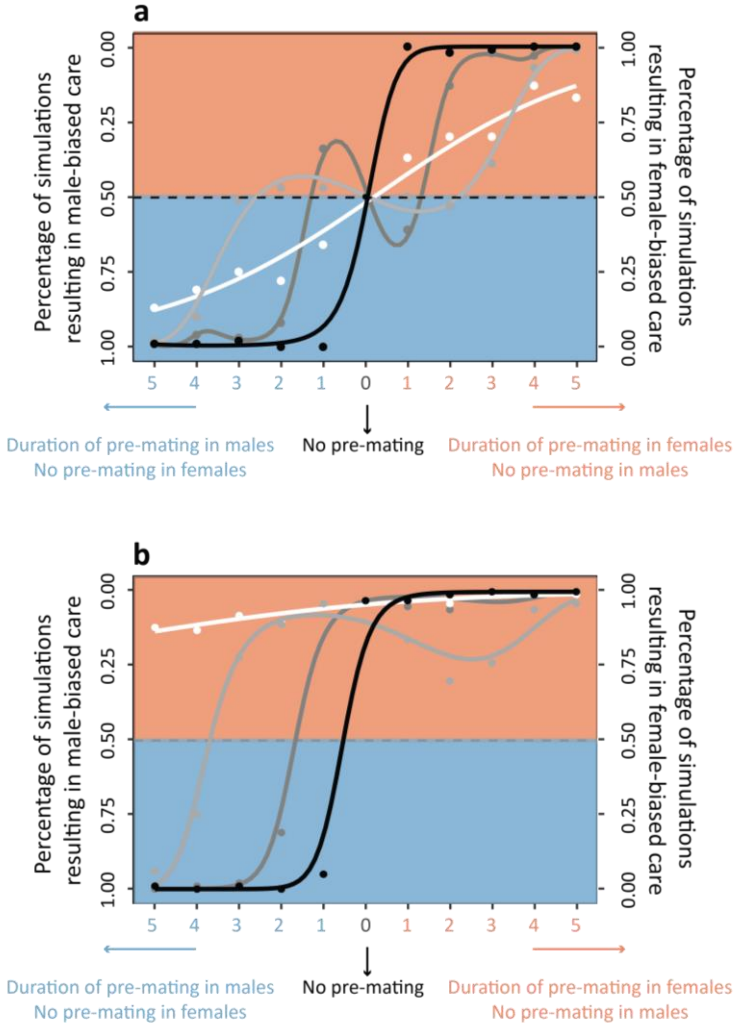

Supplementary Figure 9. Effect of sex-biased pre-mating investment and initial care conditions on parental sex roles. As shown in Fig. 7 of the main text, sex differences in pre-mating investment have a complicated effect on the evolution of parental sex roles. Here we show the effect of sex-biased pre-mating investment on parental role evolution when the population initialised with (**a**) a low egalitarian care level ($T_m = T_f = 5$), and with (**b**) different levels of female and male care ($T_f = 10$ and $T_m = 5$). In (a), the pattern is nearly identical to that in Fig. 7. Hence, the pattern in Fig. 7 seems to be robust as long as the simulations are initialised with egalitarian biparental care. However, as shown in (b), the pattern changes considerably if females and males differ in their initial care levels, especially in the case of zero mortality in the pre-mating state (white dots and line). In fact, the 'Trivers effect' completely disappeared in the case of zero mortality: the vast majority of simulations resulted in female-biased care even if the duration of the pre-mating phase was (much) longer in males than in females. When the mortality in the pre-mating period was 0.001 (light grey dots and line) or 0.002 (dark grey dots and line), female-biased care most likely evolved when the pre-mating duration was slightly male-biased; male-biased care was the more prevalent outcome only in those cases where the pre-mating duration was strongly male-biased. When the mortality in the pre-mating period was very high (0.005, black dots and line), the 'Trivers effect' was recovered: the sex with the higher pre-mating investment tended to provide more post-zygotic parental care. Note that, in the absence of pre-mating investment in either sex, the sex that initially cares more (in this case the female) is much more likely to become the caring sex in the end (in line with the findings reported in the legend of Supplementary Fig. 3). The reader should also notice that, similar to (a) and Fig. 7, the light-grey curve in (b) (corresponding to a pre-mating mortality of 0.001) is undulating, rather than monotonically increasing.

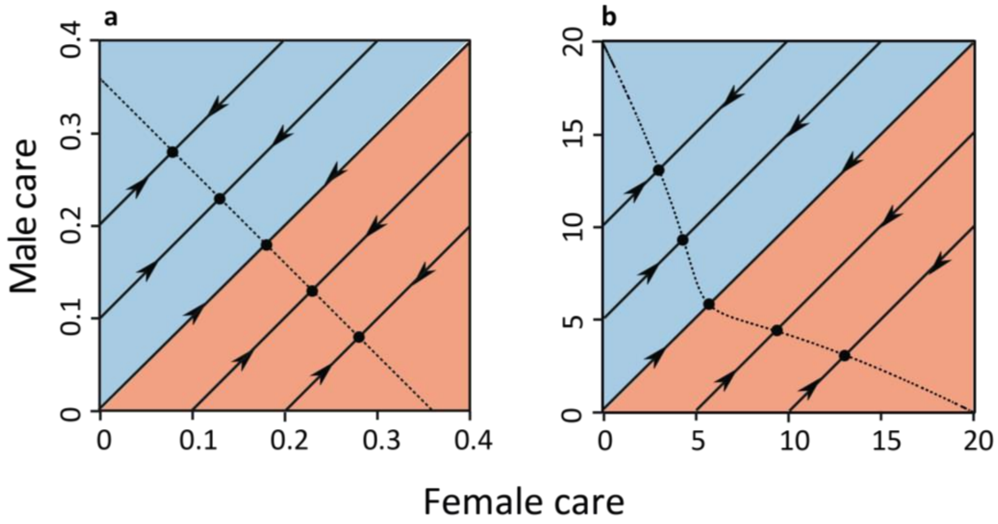

Supplementary Figure 10. Rescaled version of the Fromhage & Jennions (2016) model. The model presented here was inspired by the 'FJ model' of Fromhage and Jennions (2016, see ref. 2). However, we replaced their offspring survival function $S(T_{tot}) = \exp(-B/T_{tot})$ by the function $S(T_{tot}) = T_{tot}^2 \big/ \left(T_{tot}^2 + B^2\right)$. **(a)** For their parameter setting $B = 0.1$ and $\mu = 0.01$ (where μ denotes the mortality rate per day), Fromhage and Jennions concluded that the selection gradient method predicts convergence to a line of neutrally stable equilibria (Fig. 1a of their article). **(b)** For the parameters used in our model ($B = 20$ and $\mu = 0.001$), the FJ model produces an almost identical pattern as our variant of the model (see Fig. 2a in our main text). This shows that, also in the FJ model, the 'curve of equilibria' is not necessarily a straight line.

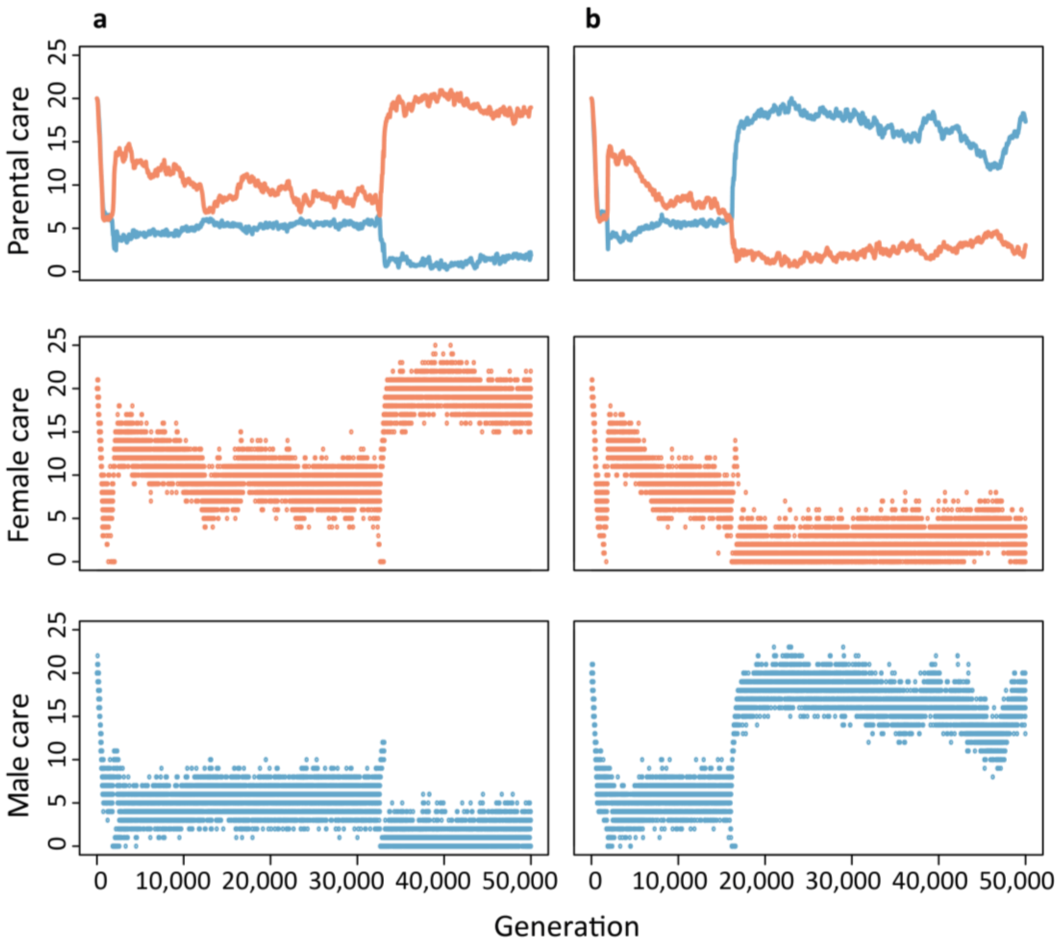

Supplementary Figure 11. Evolution of sex-biased care in the rescaled FJ model. The rescaled version of the FJ model ($B = 20$ and $\mu = 0.001$, see Supplementary Fig. 10b) exhibits a very similar behaviour as our version of the model. Two representative simulations show **(a)** the evolution of female-biased care; and **(b)** the evolution of male-biased care. On a long-term perspective, transitions between the two types of equilibria also occurred. Notice that a longer period of low-level egalitarian care precedes the first switch to sex-biased care. This is explained by the Pairwise Invasibility Plot in Supplementary Fig. 12.

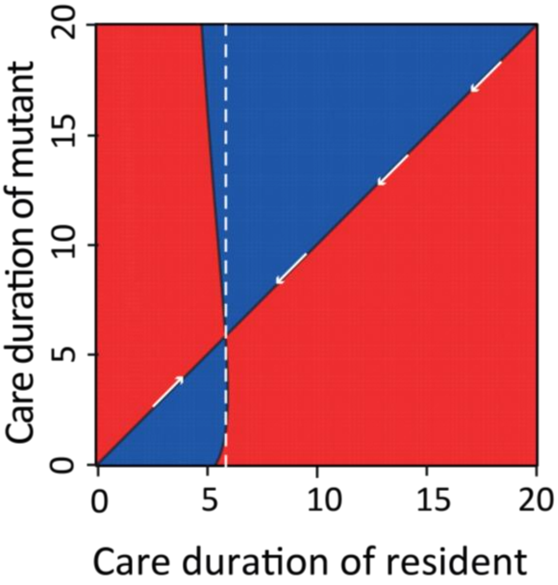

Supplementary Figure 12. Pairwise Invasibility Plot of the rescaled FJ model. With the same method as in Supplementary Fig. 2, we constructed a PIP for the rescaled FJ model (parameters $B = 20$ and $\mu = 0.001$, see Supplementary Fig. 10b). Now there is a convergence stable singular strategy at $T^* = 5.87$. In contrast to our version of the model (Supplementary Fig. 2), this singular strategy is not a branching point but evolutionarily stable (mutants close to T^* cannot invade, as the dashed vertical line lies in the blue for mutants close to T^*). Standard adaptive dynamics theory[1] would therefore predict that egalitarian care at level T^* is an evolutionary attractor, and, hence, and endpoint of evolution. In contrast, *all* simulations resulted in the evolution of sex-biased care (see Supplementary Fig. 11). Similar observations were made in other simulation studies[4,5,6], where diversification occurred at an evolutionary attractor. In all these cases, T^* is locally but not globally evolutionarily stable, as the dashed vertical line transverses the red region as well (mutants with a very short care duration can invade the population of T^* residents. If one waits long enough, such mutants will invariably appear in individual-based simulations (and in the real world). As argued in Wolf *et al.*[7], the analytical conditions for evolutionary branching are based on the assumption of infinitesimally small mutational step sizes and therefore correspond to a 'worst-case scenario for evolutionary diversification'. In simulations, diversification (or, as in our case, sex differentiation) can predictably occur under much milder conditions.

SI References

1. Geritz, S. A. H., Kisdi, E., Meszena, G. & Metz, J. A. J. Evolutionarily singular strategies and the adaptive growth and branching of the evolutionary tree. *Evol. Ecol.* **12**, 35–57 (1998).

2. Fromhage, L. & Jennions, M. Coevolutipn of parental investment and sexually selected traits drives sex-role divergence. *Nat. Commun.* **7**, 1–11 (2016).

3. Rueffler, C., van Dooren, T. J. M., Leimar, O. & Abrams, P. A. Disruptive selection and then what? *Trends Ecol. Evol.* **21**, 238–245 (2006).

4. Wolf, M., van Doorn, G. S., Leimar, O & Weissing, F. J. Life-history trade-offs favour the evolution of animal personalities. *Nature* **447**, 581–584 (2007).

5. Berngruber, T. W., Weissing, F. J. & Gandon, S. Superinfection inhibition and the evolution of viral latency. *J. Virology.* **84**, 10200–10208 (2010).

6. Baldauf, S. A., Engqvist, L. & Weissing, F. J. Diversifying evolution of competitiveness. *Nat. Commun.* **5**, 1–8 (2014).

7. Wolf, M., van Doorn, G. S., Leimar, O. & Weissing, F. J. Do animal personalities emerge? *Nature* **451**, E9–E10 (2008).
