# Supplementary material for: Transient polymorphisms in parental care strategies drive divergence of sex roles
Source: Nat Commun. 2023 Oct 26;14:6805. doi: 10.1038/s41467-023-42607-6 (PMC10603145; doi:10.1038/s41467-023-42607-6)
Supplement: Supplementary file 3 — Reporting Summary [file 41467_2023_42607_MOESM3_ESM.pdf]

nature portfolio

Corresponding author(s): Franz J. Weissing

Last updated by author(s): Sep 29, 2023

Reporting Summary

Nature Portfolio wishes to improve the reproducibility of the work that we publish. This form provides structure for consistency and transparency in reporting. For further information on Nature Portfolio policies, see our Editorial Policies and the Editorial Policy Checklist.

Statistics

For all statistical analyses, confirm that the following items are present in the figure legend, table legend, main text, or Methods section.

n/a	Confirmed	
☐	☒	The exact sample size (n) for each experimental group/condition, given as a discrete number and unit of measurement
☒	☐	A statement on whether measurements were taken from distinct samples or whether the same sample was measured repeatedly
☒	☐	The statistical test(s) used AND whether they are one- or two-sided<br>*Only common tests should be described solely by name; describe more complex techniques in the Methods section.*
☒	☐	A description of all covariates tested
☒	☐	A description of any assumptions or corrections, such as tests of normality and adjustment for multiple comparisons
☒	☐	A full description of the statistical parameters including central tendency (e.g. means) or other basic estimates (e.g. regression coefficient) AND variation (e.g. standard deviation) or associated estimates of uncertainty (e.g. confidence intervals)
☒	☐	For null hypothesis testing, the test statistic (e.g. F, t, r) with confidence intervals, effect sizes, degrees of freedom and P value noted<br>*Give P values as exact values whenever suitable.*
☒	☐	For Bayesian analysis, information on the choice of priors and Markov chain Monte Carlo settings
☒	☐	For hierarchical and complex designs, identification of the appropriate level for tests and full reporting of outcomes
☒	☐	Estimates of effect sizes (e.g. Cohen's d, Pearson's r), indicating how they were calculated

Our web collection on statistics for biologists contains articles on many of the points above.

Software and code

Policy information about availability of computer code

Data collection	Visual Studio Community 2019 on Windows, XCode 15 on Mac or G++ Compiler on Linux can be used to compile the C++ code for individual-based simulations. Mathematica (12.0.0) was used to conduct all mathematical analyses.
Data analysis	All simulation data were analysed in R (version 4.3.1). R-packages used are shape, dplyr, stringr, tidyverse, readr, gridExtra, gtools and ggplot2.

For manuscripts utilizing custom algorithms or software that are central to the research but not yet described in published literature, software must be made available to editors and reviewers. We strongly encourage code deposition in a community repository (e.g. GitHub). See the Nature Portfolio guidelines for submitting code & software for further information.

Data

Policy information about availability of data

All manuscripts must include a data availability statement. This statement should provide the following information, where applicable:

- Accession codes, unique identifiers, or web links for publicly available datasets
- A description of any restrictions on data availability
- For clinical datasets or third party data, please ensure that the statement adheres to our policy

All simulated data that were used to generate figures in this study, the C++ code for individual-based simulations, the R-script for data analysis and the Mathematica file for mathematical analysis are available for download from https://zenodo.org/record/8114131.

March 2021

Human research participants

Policy information about studies involving human research participants and Sex and Gender in Research.

Reporting on sex and gender	No human data for this study.
Population characteristics	No human data for this study.
Recruitment	No human data for this study.
Ethics oversight	No human data for this study.

Note that full information on the approval of the study protocol must also be provided in the manuscript.

Field-specific reporting

Please select the one below that is the best fit for your research. If you are not sure, read the appropriate sections before making your selection.

☐ Life sciences ☐ Behavioural & social sciences ☒ Ecological, evolutionary & environmental sciences

For a reference copy of the document with all sections, see nature.com/documents/nr-reporting-summary-flat.pdf

Ecological, evolutionary & environmental sciences study design

All studies must disclose on these points even when the disclosure is negative.

Study description	Animal species differ a lot in how the parental tasks are distributed between the male and female parent. To understand what drives this diversity, we closely inspected the evolution of sex-specific parental behaviour in computer simulations. We found that pronounced sex differences (either female-biased care or male-biased care) rapidly evolve, even if such differences are initially absent. Our conclusions differ strongly from those of the mathematical models that form the basis of standard sex role theory. To uncover the reason for this, we systematically investigate our model from both a simulation and a mathematical perspective. It turns out that the discrepancies are caused by the fact that in the simulations sexual conflict drives the emergence of within-sex polymorphisms in parental care strategies, which in turn, drive the evolution of asymmetry in parental care between the two sexes in a characteristic and highly repeatable manner.
Research sample	All data were generated by running computer simulations. At least 100 replicate simulations were run for each parameter setting.
Sampling strategy	For each parameter setting, we ran at least 100 replicate simulations. No statistical strategy is used to determine the number of simulations.
Data collection	All data were collected from the computer simulations. For our evolving parameters (female care, male care, female preferences and male ornaments), both the population average and individual data are collected.
Timing and spatial scale	Each simulation was run for at least 5,000 generations(= 5,000,000 time steps). The model is not spatially explicit.
Data exclusions	No data were excluded from the analyses. For visualization purposes, randomly chosen simulations were represented, and subset of the data were shown in the figures. Our selection of data presented is justified in the accompanying text.
Reproducibility	Simulations are replicable when using the same parameter settings.
Randomization	A random seed was generated for each simulation.
Blinding	Blinding was not performed as the data were analyzed algorithmically; results for each simulation were processed using the same computational pipeline.

Did the study involve field work? ☐ Yes ☒ No

Reporting for specific materials, systems and methods

We require information from authors about some types of materials, experimental systems and methods used in many studies. Here, indicate whether each material, system or method listed is relevant to your study. If you are not sure if a list item applies to your research, read the appropriate section before selecting a response.

Materials & experimental systems

n/a	Involved in the study	
☒	☐	Antibodies
☒	☐	Eukaryotic cell lines
☒	☐	Palaeontology and archaeology
☒	☐	Animals and other organisms
☒	☐	Clinical data
☒	☐	Dual use research of concern

Methods

n/a	Involved in the study	
☒	☐	ChIP-seq
☒	☐	Flow cytometry
☒	☐	MRI-based neuroimaging
